# Unveiling the Antiviral Efficacy of Forskolin: A Multifaceted In Vitro and In Silico Approach

**DOI:** 10.3390/molecules29030704

**Published:** 2024-02-03

**Authors:** Yhiya Amen, Mohamed A Selim, Reda A. Suef, Ahmed M. Sayed, Ahmed Othman

**Affiliations:** 1 Department of Pharmacognosy, Faculty of Pharmacy, Mansoura University, Mansoura 35516, Egypt; 2 Botany and Microbiology Department, Faculty of Science, Al-Azhar University, Cairo 11884, Egypt; mohamedselim@azhar.edu.eg (M.A.S.); redasuef@azhar.edu.eg (R.A.S.); 3 Department of Pharmacognosy, Collage of Pharmacy, Almaaqal University, Basrah 61014, Iraq; ahmed.mohamed.sayed@nub.edu.eg; 4 Department of Pharmacognosy, Faculty of Pharmacy, Nahda University, Beni-Suef 62513, Egypt; 5 Department of Pharmacognosy and Medicinal Plants, Faculty of Pharmacy, Al-Azhar University, Cairo 11884, Egypt

**Keywords:** *Coleus forskohlii*, Forskolin, HAV, COX-B4, HSV-1, HSV-2, virtual screening, MD simulation, Cathepsin L

## Abstract

*Coleus forskohlii* (Willd.) Briq. is a medicinal herb of the Lamiaceae family. It is native to India and widely present in the tropical and sub-tropical regions of Egypt, China, Ethiopia, and Pakistan. The roots of *C. forskohlii* are edible, rich with pharmaceutically bioactive compounds, and traditionally reported to treat a variety of diseases, including inflammation, respiratory disorders, obesity, and viral ailments. Notably, the emergence of viral diseases is expected to quickly spread; consequently, these data impose a need for various approaches to develop broad active therapeutics for utilization in the management of future viral infectious outbreaks. In this study, the naturally occurring labdane diterpenoid derivative, Forskolin, was obtained from *Coleus forskohlii*. Additionally, we evaluated the antiviral potential of Forskolin towards three viruses, namely the herpes simplex viruses 1 and 2 (HSV-1 and HSV-2), hepatitis A virus (HAV), and coxsackievirus B4 (COX-B4). We observed that Forskolin displayed antiviral activity against HAV, COX-B4, HSV-1, and HSV-2 with IC_50_ values of 62.9, 73.1, 99.0, and 106.0 μg/mL, respectively. Furthermore, we explored the Forskolin’s potential antiviral target using PharmMapper, a pharmacophore-based virtual screening platform. Forskolin’s modeled structure was analyzed to identify potential protein targets linked to its antiviral activity, with results ranked based on Fit scores. Cathepsin L (PDB ID: 3BC3) emerged as a top-scoring hit, prompting further exploration through molecular docking and MD simulations. Our analysis revealed that Forskolin’s binding mode within Cathepsin L’s active site, characterized by stable hydrogen bonding and hydrophobic interactions, mirrors that of a co-crystallized inhibitor. These findings, supported by consistent RMSD profiles and similar binding free energies, suggest Forskolin’s potential in inhibiting Cathepsin L, highlighting its promise as an antiviral agent.

## 1. Introduction

The utilization of arrays of natural bioactive compounds, especially those from traditional medicines in the exploration of new drugs, presents a number of benefits that contribute to their ongoing importance in pharmaceutical research and development. Some of the major advantages include both chemical structures and biological activities [1], biologically validated interactions with modulating enzymes and different proteins [2], historical significance spanning centuries, with roughly 50% of today’s pharmaceuticals derived from natural products [3], and serving as a source for novel bioactive compounds with a range of chemical compositions that can be utilized in treatments of different diseases [4]. The World Health Organization (WHO) reports documented around a total of 374,000 plants [5], with 28,187 of them being utilized by humans for therapeutic purposes [6]. Furthermore, the antibacterial activity of about 1340 plants has been stated, with over 30,000 biologically active antimicrobial compounds originating from plants [7]. This highlights the promising potential of medicinal plants in developing pharmaceutical candidates with antimicrobial characteristics.

Viral infections are currently regarded as the most significant threat among all historical outbreaks of infectious diseases in recent times [8]. Their variable replicative modes and transmission patterns, alongside the lack of targeted broad-spectrum antiviral approaches that minimize interference with host cell functions, poses a significant challenge in combating viral infections [8]. Antiviral synthetic medications have been employed to suppress virus replications through diverse mechanisms [9]. However, challenges in antiviral medications emerge due to their limited cost and cost effectiveness, potential toxicity/side effects, and the emergence of viral resistance [10], which confirm the importance of assessing the antiviral medicinal significance of different plant-based alternatives, particularly in light of limited research on plant-derived antiviral compounds when compared to intensive studies on antimicrobial compounds [11].

*Coleus forskohlii* is a tropical perennial plant that belongs to the mint family, *Lamiaceae*, a native indigenous India, Nepal, and Thailand source. *Coleus forskohlii* has been used for centuries in traditional medicine systems, particularly in Ayurveda, and pharmaceutical and food industries, with, for example, edible tubers being used for pickles [12]. Forskolin (7-β-acetoxy-8, 13-epoxy-1 β, 6 β, 9 β -trihydroxy-labd-14-ene-11-one) is a labdane diterpene (the exclusive alkaloid Forskolin source of the plant kingdom) [13], with the chemical formula C_22_H_34_O_7_, and it is the primary active ingredient present in the Coleus plant’s root cork tissue (*Coleus forskohlii).* In traditional Indian medicine, this unique biocompound has a broad-spectrum pharmacological potential for treating conditions such as glaucoma, tumors, HIV, obesity, hypertension, heart diseases, asthma, and cardiac complications [14]. This is due to the activation utility of adenylate cyclase, leading to the modulation of intracellular cyclic adenosine monophosphate (cAMP) [15] levels, which in turn triggers various cellular responses and signaling pathways.

Forskolin has been reported as a promising antiviral agent. In silico analyses have suggested that Forskolin could potentially inhibit SARS-CoV-2; specifically, Forskolin was docked with the ACE2 receptor, which is the binding target of the S1 unit of the viral S protein of SARS-CoV-2. The in silico docking studies indicated that Forskolin may play a crucial role in inhibiting the protein–protein interaction of the receptor-binding domain (RBD) and ACE2, potentially preventing the entrance of SARS-CoV-2 into human cells for infection [16]. Furthermore, Forskolin has been reported to have a role as an anti-HIV agent and a protein kinase A agonist [17]. While these findings show promise, it is important to acknowledge that the supporting evidence for Forskolin as an antiviral agent is still limited and inconclusive. Further research, including in vitro studies, is necessary to assess its efficacy and safety against different types of viruses.

The hepatitis A virus (HAV), the herpes simplex virus type 1 and 2 (HSV-1 and 2), and coxsackievirus B4 (CoxB4) are responsible for a range of life-threatening viral infections. HSV are commonly and globally spreading viral infections, affecting approximately 67% and 13% of the global population, respectively. In 2016, it was estimated that around 3.7 billion individuals were seropositive for HSV-1 and nearly 500 million for HSV-2 [18]. These viruses can be transmitted through close contact, leading to a lifelong dormant infection. HSV-1 is typically acquired during early childhood through the orolabial mucosa, while HSV-2 infections often occur later in life via sexual transmission. Infection with HSV causes cold sores, genital herpes, herpes stromal keratitis, eczema herpeticum, disseminated disease in newborns, meningitis, herpes simplex encephalitis, as well as the reported correlation between HSV and neurodegenerative disorders [19].

Infection with HAV is highly transmissible [20] and is a major contributor of acute hepatitis cases. Among individuals with underlying liver conditions, acute HAV infection can trigger liver failure [21]. The prevalence of HAV antibodies in high-income regions tends to be relatively low due to socioeconomic factors, access to clean water, and proper sanitation practices [22]. Generally, HAV causes a short-term illness that usually resolves within 4–7 weeks without long-term consequences. Unlike HBV and HCV, persistent liver damage does not appear to occur as a result of HAV infection. However, severe fulminant hepatitis, leading to potentially fatal liver failure, may develop in older individuals [23].

Coxsackieviruses B are a type of enterovirus categorized under the *Picornaviridae* family. The genus enterovirus consists of 12 species, namely enterovirus A–D and rhinovirus A–C, with their classification determined by genetic identification. Within Enterovirus B, more than 63 serotypes including CV-B 1-6 as well as echoviruses are present. CV-B infections result in spastic paralysis and can target various tissues within the human body, such as the central nervous system, liver, endocrine and exocrine pancreas, brown fat tissue, along with striated muscle cells [24].

The major challenge in the treatment of those viruses is the emergence of antiviral drug resistance during infections, emphasizing the need for new and effective therapies for prophylaxis and the treatment of viral infections. Therefore, our study aims to evaluate the antiviral effects of Forskolin against different types of viruses, including the life-threatening HSV, the highly contagious HAV, and Cox-B4 virus, which is a crucial for expanding our knowledge of natural compounds with potential antiviral properties and may pave the way for the development of novel antiviral therapies.

## 2. Results and Discussion

### 2.1. Identification of the Isolated Compound

**Figure 1:**
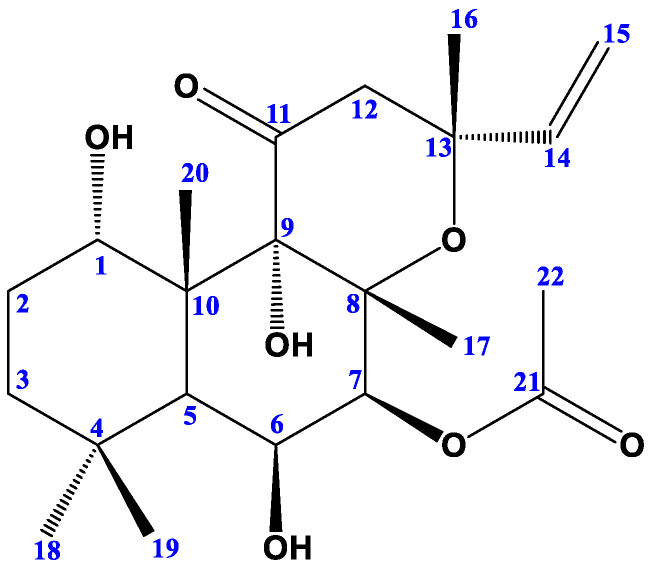
Structure of Forskolin.

The HR-ESI-MS spectrum showed a sodiated molecular ion peak at *m*/*z* 433.2173 [M + Na]^+^, calculated (433.2202) and a deprotonated molecular ion peak at *m*/*z* 409.2237 [M − H]^−^, calculated (409.2226) in accordance with the molecular formula C_22_H_34_O_7_. The ^1^H-NMR spectrum of compound C1 (Forskolin) revealed the presence of five singlets assigned for five tertiary methyl groups at δ_H_ 1.04 (3H-18), 1.26 (3H-19), 1.34 (3H-16), 1.44 (3H-20), and 1.71 (3H-17). In addition to the sixth CH_3_ group at δ_H_ 2.16 (3H, s, OCOCH_3_), it also displayed a double doublet at δ_H_ 5.87 (1H, dd, *J* = 10.6, 17.2 Hz), assigned for olefinic proton H-14, corresponding to C-14 (δ_C_ 146.3). The splitting pattern clearly showed coupling between H-14 and the two protons resonated at δ_H_ 4.91 (1H, d, *J* = 10.9) and 5.40 (1H, d, *J* = 17.2), which are assigned for 2H-15, and corresponding to the olefinic methylene C-15 (δc 110.7). The presence of the three signals at δ_H_ 4.5 (1H, brs, H-6), 4.6 (1H, brs, H-1), and 5.28 (1H, H-7, d, *J* = 16.3) with corresponding δ_C_ 70.0 (C-6), 74.4 (C-1), and 76.5 (C-7) confirmed that these three methines are oxygenated. Additionally, the ^13^C-NMR spectrum revealed the presence of 22 signals, and their assignments are shown in Table S1. Consequently, compound **C1** was identified as Forskolin (Figure 1).

### 2.2. Biological Evaluation

In the current study, the isolated compound was tested for its antiviral activities against herpes simplex viruses 1 and 2 (HSV-1 and HSV-2), hepatitis A virus (HAV), and coxsackievirus B4 (COX-B4).

#### 2.2.1. Cytotoxicity Assay

The cytotoxic properties of the Forskolin extract were evaluated on Vero cell lines by treating them for 48 hours with decreasing extract concentrations ranging from 1000 to 31.25 μg/mL using the MTT cell proliferation assay. The results showed a dose-dependent cytotoxic response, with a mild reduction in cell growth observed at lower extract concentrations (ranging from 125 to 31.25 μg/mL). However, at higher doses of the extract (up to 1000 μg/mL), the inhibitory effect was stronger, indicating the concentration-dependent effect of the extract on cell growth inhibition. The CC_50_ values were determined to be 322.1 μg/mL, with MNTC values of 125 μg/mL (Table S2; Figure S6). Therefore, the relationship between CC_50_ and MNTC indicates that the extract’s cytotoxic effects are observed at higher concentrations (CC_50_ = 322.1), while lower concentrations (MNTC = 125) do not cause any harm to the cells. This information highlights the importance of determining the appropriate concentration range for studying the antiproliferative effects of the extract.

#### 2.2.2. Vero Cell Lines Morphological Changes

**Figure 2:**
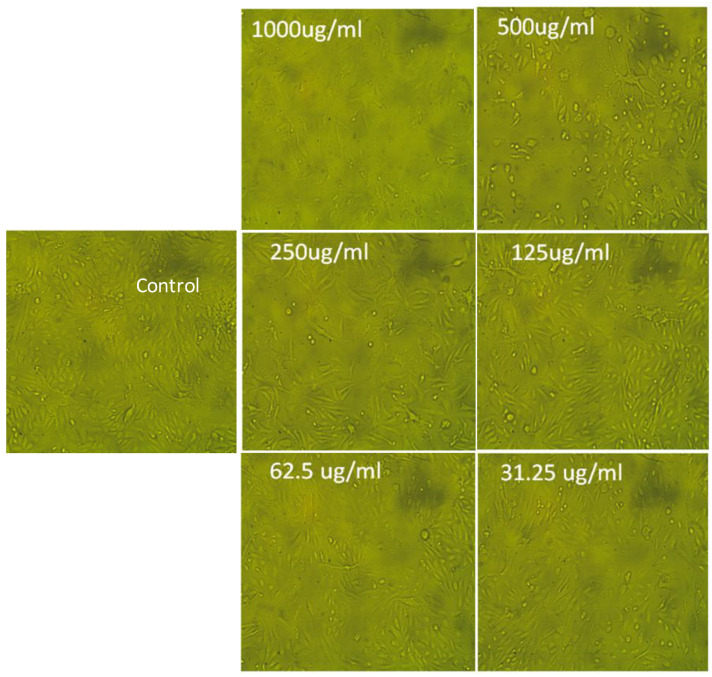
Morphological changes examination of Vero cell lines using a phase-contrast light inverted microscope, after 48 h of culturing with different concentrations of Forskolin, in comparison to the control. The magnification power is 100X.

The changes in the morphological appearance of Vero cell lines observed at different Forskolin concentrations were reported as a significant reduction in cell number and size after treatment with the extract at the highest concentrations (1000 µg/mL) tested (Figure 2).

#### 2.2.3. Antiviral Activity of Forskolin (Detection of IC_50_)

Forskolin was assessed for its antiviral activity against herpes simplex viruses 1 and 2 (HSV-1 and HSV-2), hepatitis A virus (HAV), and coxsackievirus B4 (COX-B4) using Vero cell lines. The isolated Forskolin exhibited relatively low inhibitory concentration 50 (IC_50_) values, indicating possible antiviral activities against the four investigated viruses, as seen in Table 1. Forskolin displayed the highest antiviral activity against HAV and COX-B4, with IC_50_ values of 62.9 and 73.1 μg/mL, respectively. Additionally, Forskolin showed antiviral activity against HSV-1 and HSV-2, with IC_50_ values of 99.0 and 106.0 μg/mL, respectively.

### 2.3. In Silico Studies

#### 2.3.1. Virtual Screening-Based Target Identification

**Figure 3:**
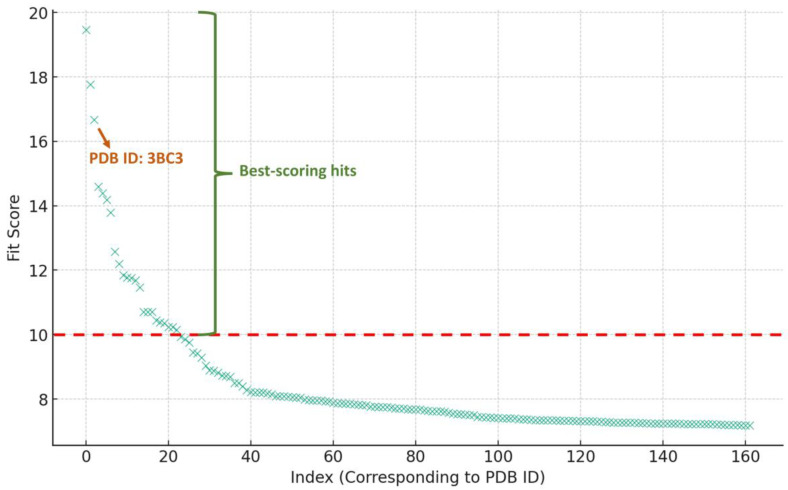
The PharmMapper results are illustrated in a scatter-plot, showcasing the potential protein targets of Forskolin, along with their respective fit scores. A threshold fit score of 10 has been established as the criterion for selection. Among the highest-scoring hits, Cathepsin L, identifiable by its PDB ID 3BC3 and boasting a fit score of 16.8, emerged as the sole protein target relevant to antiviral activity.

In order to putatively characterize the antiviral-relevant target of Forskolin, we used a pharmacophore-based virtual approach using the PharmMapper platform [25]. PharmMapper can screen and suggest the most likely protein targets of a query molecule, based on its pharmacophore model by mapping its key pharmacophore features (i.e., spatial arrangement of structural features). Accordingly, molecules that conform to these pharmacophore maps have a greater potential for binding to the same protein targets. Therefore, the modeled structure of Forskolin was analyzed using PharmMapper to identify the potential protein target(s) that may be associated with its antiviral activity. The results that were retrieved were ranked by how well they fit to the protein target (i.e., Fit score; Figure 3). Only viral targets were selected, particularly those relevant to the tested strains. Among the best-scoring hits (i.e., hits with Fit scores higher than 10), Cathepsin L (PDB ID: 3BC3; Fit score = 16.8, Figure 3), [26] was the only target relevant to antiviral activity. As a result, Cathepsin L was selected for the subsequent in silico experiments.

Cathepsin L, a lysosomal cysteine protease, plays a crucial role in viral infections, acting as a facilitator in the life cycle of various viruses [27,28]. In the context of herpes simplex viruses 1 and 2 (HSV-1 and HSV-2), Cathepsin L is instrumental in the processing of viral glycoproteins, which are essential for the viruses’ entry into host cells. This protease specifically cleaves and activates these glycoproteins, thus aiding in the fusion of the viral envelope with the host cell membrane, a key step in viral entry [29,30]. Similarly, in Hepatitis A virus (HAV) infections, Cathepsin L is involved in the disassembly and release of the viral genome into the cytoplasm of the host cell. This process is critical for the initiation of viral replication [31,32]. Furthermore, in the case of Coxsackievirus B4 (COX-B4), Cathepsin L has been implicated in the uncoating of the virus, a process that releases the viral RNA into the host cell, thereby facilitating the subsequent stages of the viral life cycle [33,34]. Understanding the role of Cathepsin L in these viral infections not only sheds light on the intricate mechanisms of viral pathogenesis, but also opens avenues for developing targeted antiviral therapies that can disrupt these key interactions.

Building on the understanding of Cathepsin L’s role in the life cycle of various viruses, it becomes evident that targeting and inhibiting this protease could yield a broad-spectrum antiviral strategy. Since Cathepsin L is a common denominator in crucial steps like viral entry, genome uncoating, and replication for diverse viruses such as HSV-1, HSV-2, HAV, and COX-B4, its inhibition could disrupt these critical phases, effectively impeding the progression of these infections. This approach of targeting a host cell factor, rather than the virus itself, also offers an advantage in combating viral resistance, a common challenge with traditional antiviral drugs that target viral components. Furthermore, given the wide range of viruses that rely on Cathepsin L, inhibitors designed against this protease hold the potential for broad-spectrum efficacy, transcending the limitations of virus-specific drugs [35,36,37]. Therefore, this strategy not only promises a powerful tool in the fight against known viruses but also offers a robust platform to counter emerging viral threats, thereby representing a significant leap forward in antiviral therapy.

#### 2.3.2. Molecular Docking

To explore how Forskolin interacts with Cathepsin L, their structural models were prepared, undergoing a molecular docking simulation. This was followed by assessing 10 retrieved binding poses (Figure S8, Table S8) through short molecular dynamic (MD) simulations lasting 30 nanoseconds; this was aimed at confirming the most stable binding pose within the enzyme’s active site. The top-scoring docking pose, with a score of −10.37 kcal/mol, demonstrated the greatest stability, evidenced by the lowest root-mean-square deviation (RMSD) throughout the simulation, averaging at 1.87 Å. Consequently, this binding pose was chosen for an extended 200 ns MD simulation in order to delve deeper into the dynamics of Forskolin’s binding within Cathepsin L’s active site.

#### 2.3.3. Molecular Dynamics Simulation

**Figure 4:**
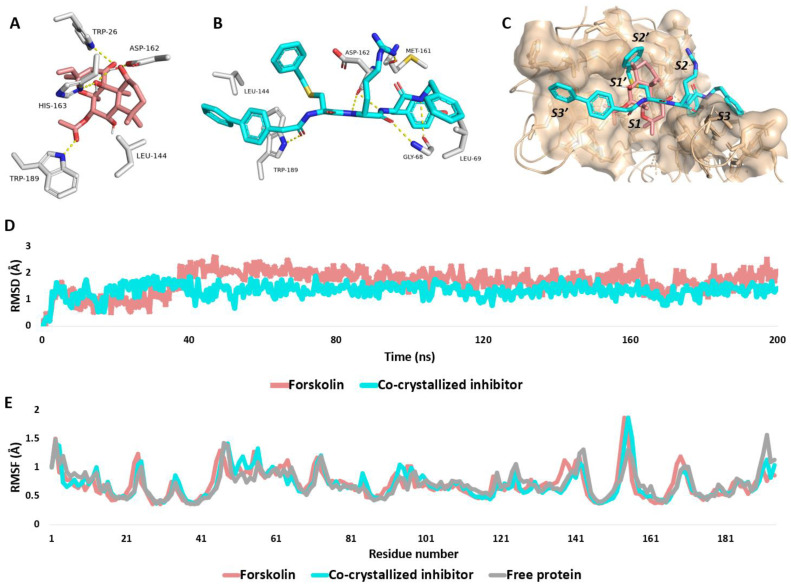
(**A**,**B**): Dynamic binding modes of Forskolin and the co-crystallized inhibitor, respectively, inside the active site of Cathepsin L (PDB ID 3BC3). (**C**): Binding modes of Forskolin (brick red-colored structure) in alignment with the co-crystalized inhibitor. Forskolin occupied both S1 and S1′ subsites of cathepsin L’s active site. (**D**): The RMSDs of both Forskolin and the co-crystalized inhibitor inside the binding site of Cathepsin L over the course of 200 ns-long MD simulation. (**E**): RMSF profile of cathepsin L in its free unliganded form and in complex with both Forskolin and the co-crystallized inhibitor.

As illustrated in Figure 4, the RMSD profiles of Forskolin and the co-crystallized inhibitor were strikingly similar, both displaying an average RMSD of around 1.98Å. This consistent binding was reflected in their comparable absolute binding free energies, with values of −8.86 and −9.47 kcal/mol, respectively. Regarding the impact of Forskolin binding, our RMSF analysis of Cathepsin L revealed that the binding of Forskolin, as well as the co-crystallized inhibitor, did not induce significant alterations in the protein’s dynamics, as depicted in Figure 4E. This observation suggests a preservation of structural stability, including the active site, upon ligand interaction.

Upon the further analysis of their dynamic binding modes, it was observed that the modeled structure of Forskolin achieved a binding mode somewhat similar to that of the co-crystallized inhibitor, effectively occupying the S1 and S1′ subsites of the active site (Figure 4C). Hydrogen bonding was the predominant interaction within the enzyme’s active site, where Forskolin’s modeled structure formed stable hydrogen bonds with ASP-162 and TRP-189, akin to the co-crystallized inhibitor, and additional bonds with TRP-26 and HIS-163. In terms of hydrophobic interactions, Forskolin’s structure showed stable interactions with LEU-144, similar to the co-crystallized inhibitor.

**Figure 5:**
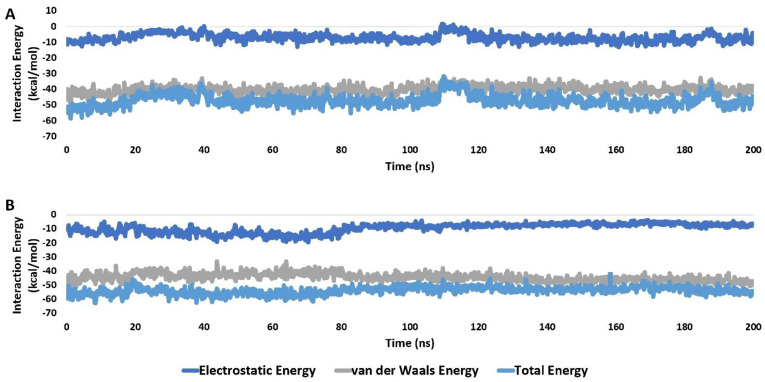
Interaction energies (i.e., electrostatic and van der Waals interaction energies) of Forskolin and the co-crystallized inhibitor (A and B, respectively) inside the active site of cathepsin L (PDB ID 3BC3) over the course of a 200 ns−long MD simulation.

This stable binding mode resulted in significant and stable interaction energies (electrostatic and van der Waals) within the Cathepsin L binding site. Both Forskolin and the co-crystallized inhibitor displayed average total interaction energies of around −51.11 and −58.43 kcal/mol, respectively, as shown in Figure 5.

Considering these modeling and MD simulation results, it can be inferred that Forskolin likely exerts its antiviral effects by targeting Cathepsin L.

It is worth noting that, in the realm of computational drug discovery, the virtual screening process stands as a cornerstone, offering predictions of potential drug-target interactions based on algorithmic simulations. However, this study has highlighted the nuanced and sometimes unpredictable nature of such computational methods, exemplified by the discrepancy in the identification of Cathepsin L as a target in independent PharmMapper screenings. This inconsistency is not merely an anomaly, but rather an insightful observation, prompting a deeper examination of the factors influencing virtual screening outcomes.

One of the pivotal elements under scrutiny is the inherent variability of results rendered by online-based computational platforms like PharmMapper. These platforms, while powerful, are subject to the dynamism of computational algorithms, which can exhibit non-deterministic characteristics, due to the complex interplay of numerous variables. A notable factor is the server load at the time of query processing, which can significantly impact the computational resources allocated for each screening, thereby influencing the depth of the screening process and the accuracy of the results. Such variations in computational resource allocation underscore the susceptibility of virtual screening outcomes to external computational conditions.

Moreover, the initial geometric conformation of the query molecule plays an instrumental role in shaping the interaction landscape explored during the virtual screening process. Even minor deviations in the three-dimensional structure can lead to markedly different sets of predicted binding affinities and potential targets. This study’s findings, particularly the absence of Cathepsin L in the list of targets from one of the PharmMapper runs, exemplify how subtle differences in the query molecule’s conformation can lead to significant disparities in the predicted interaction profiles.

Acknowledging these challenges, this research did not solely rely on the initial output from PharmMapper. Instead, it adopted a multi-faceted computational approach, integrating redocking, binding-free energy calculations, and molecular dynamic (MD) simulations. This comprehensive strategy aimed to refine the initial screening results, providing a more accurate, reliable, and holistic understanding of the interaction landscape. By doing so, this study not only addresses the inherent uncertainties associated with individual computational techniques, but also enhances the robustness and reproducibility of the findings, setting a precedent for future computational drug discovery endeavors.

In conclusion, the observed discrepancy in the identification of Cathepsin L underscores the intricate and dynamic nature of computational drug discovery. It serves as a reminder of the importance of a meticulous, multi-tiered computational approach, especially when navigating the complex and sometimes unpredictable terrain of virtual screening. Through such diligent and integrative methodologies, the field can continue to advance, harnessing the full potential of computational tools to unravel the complexities of drug–target interactions.

## 3. Materials and Methods

### 3.1. General Experimental Procedures

Silica gel (75–120 mesh), RP-C_18_ silica (38–63 μm), and organic solvents were purchased from Wako Pure Chemical Industries (Osaka, Japan). Sephadex LH-20 was purchased from GE Healthcare (Uppsala, Sweden). Analytical TLC was performed on precoated silica gel 60 GF_254_ (20 × 20 cm × 0.2 mm thick) or precoated RP-C_18_ F_254_ plates (5 × 7.5 cm × 0.2 mm thick) on aluminium sheets, from Merck Co., Darmstadt, Germany. Ultraviolet (UV) spectra were obtained using UV-visible spectrophotometer (Shimadzu 1601 PC, model TCC240, Kyoto, Japan). Optical rotations were measured with a Jasco DIP-370 polarimeter. Then, 1D and 2D spectra were obtained using a Bruker DRX 600 NMR spectrometer (Bruker Daltonics Inc., Billerica, MA, USA), using TMS as an internal standard. Mass analysis (HRMS) was performed with an Agilent 6545 Q-TOF mass spectrometer (Agilent Technologies, Santa Clara, CA, USA).

### 3.2. Plant Material

The ethanolic extract of *Coleus forskohlii* root powder [Batch C181225EM] was kindly provided by the Sabinsa Japan Corporation, Tokyo, Japan. 

### 3.3. Extraction and Isolation Procedures

For this process, 50 g of the ethanolic extract was dissolved in a minimum amount of MeOH in a separating funnel, diluted with 250 mL of distilled water and then extracted with dichloromethane (DCM). The solvent was evaporated under reduced pressure to give 33.2 g DCM-soluble fraction. Then, 1 g of DCM-soluble fraction was chromatographed on MPLC Pure C-850 Flash prep^®^ (Buchi, Flawil, Switzerland) using *n*-hexane: EtOAc (100:0 → 0:100) on a prepacked normal phase flash column (12 g), and a flow rate of 30 mL/min. using ELSD and DAD (200, 254 nm) detectors to give 59 subfractions (subfractions 1–59). Subfractions (17–22; 185.5 mg), eluted with 10% EtOAc/*n*-Hexane, were further chromatographed on MPLC Pure C-850 Flash prep^®^ using *n*-hexane: EtOAc (100:0 →80:20) on a prepacked normal phase flash column (12 g), and a flow rate of 20 mL/min. using ELSD detector to give 75 subfractions (subfractions 1–75). Subfractions 45–56 were further purified by repeated crystallization from DCM-MeOH (90:10) to give compound **C1** (119.8 mg) as off-white shiny flakes.

### 3.4. Reagents for Biological Assay, Cell Lines, and Experimental Strains

RPMI-1640 medium, DMEM, fetal bovine serum (FBS), penicillin–streptomycin, Trypan-blue dye, dimethyl sulfoxide (DMSO), 3-(4,5-dimethylthiazol-2-yl)-2,5-diphenyl tetrazolium bromide (MTT) were provided from the Faculty of Medicine for Girls, Al-Azhar University, Cairo. A green monkey kidney cell line (Vero) was utilized in our study (obtained from the American Type Culture Collection). The cells were cultured in completed RPMI-1640 media with 10% FBS and 1% penicillin–streptomycin. Vero cells are maintained in a humidified incubator at 37 °C and 5% CO_2_ atmosphere with 95% humidity. The experimental strains of the viruses were HSV1, HSV2, CoxB4, and HAV, which were provided by the Faculty of Medicine for Girls, Al-Azhar University, Cairo, Egypt.

### 3.5. Preparation of Forskolin Dilutions

A series dilutions of Forskolin extract were prepared in DMEM at dilution with differing concentrations (1000, 500, 250, 125, 62.5, 31.25 µg/mL).

### 3.6. MTT Cell Viability and Cytotoxicity Assessment

The toxicity of Forskolin and cell viability, at tested concentrations, was evaluated on Vero cell lines, using the MTT cell viability assay [38]. The mean inhibitory concentration (IC_50_) and the maximum non-toxic concentration [MNTC] of Forskolin on Vero cell lines were calculated. Briefly, the cells were maintained in a 5% CO_2_ incubator, cultured in a RPMI-1640 complete medium, supplemented with 10% (*v*/*v*) fetal bovine serum at 37 °C. After forming a confluent sheet of Vero cells, the growth medium was removed from the microtiter plates, and the cell monolayer was washed twice with wash media. The cells were then seeded (at a concentration of (1 × 10^4^ cells/well) into 96-well cell culture plates and incubated for 24 h in RPMI-1640 complete medium. Subsequently, cells were incubated at 37 °C for up to two days with 2 mL of Forskolin at the varying prepared concentrations (1000, 500, 250, 125, 62.5, 31.25 µg/mL) in triplicates. The cells were examined for any physical signs of toxicity such as the partial or complete loss of the monolayer, rounding, shrinkage, or cell granulation. After 48 h, Vero cells of 96-well plates, the supernatants were removed and subsequently, 20 µL of the MTT solution (5 mg/mL in PBS) was added each well. The plate was then placed on a shaking table at 150 rpm for 5 min to ensure thorough mixing. Following this, the plate was incubated at 37C with 5% CO_2_ for 4 h. The supernatants were removed and 200 µL of DMSO was added to each well to dissolve the produced formazan crystals. This mixture was subjected to further shaking on a table set at 150 rpm for another 6 min to fully integrate solvent with the formed formazan. The quantitation of MTT reduction was measured at 560 nm absorbance with background removal at 620 nm, using microplate ELISA. The cell viability percentage (%) was determined using the following formula: the viability (%) = [Absorbance of treated wells / Absorbance of control wells] × 100.

### 3.7. Forskolin Antiviral Activities Assessment

The Vero cell lines were cultured in a complete Dulbecco’s modified Eagle’s medium (DMEM; Gibco/Invitrogen, Carlsbad, CA, USA) with 10% fetal bovine serum (heat inactivated, Gibco) with 5% CO_2_ at 37 °C. The culture medium was changed every other day, and cell growth was monitored daily. Following the collection of the virus stock solution, the TCID50 values for the targeted viruses at different concentrations of Forskolin were calculated. The experimental strains of the viruses were HSV1, HSV2, CoxB4, and HAV. The antiviral test was carried out in 96-well cell culture plates. The Vero cells of 96-well plates (1 × 10^4^ = 200 μL cells/well) were seeded (treated) with an equal volume (100 µL = 1:1 *v*/*v*) of non-lethal dilution of tested crude plant extracts, and the targeted virus (HSV1, HSV2, CoxB4, and HAV) suspensions (serial dilution 125, 62.5, 31.25, 15.62 mg/mL) were added for one hour under shaking table conditions of 150 rpm for five minutes. This was followed by virus-cell-Forskolin incubation at 37 °C in the presence of 5% CO_2_ for 48 h. The supernatant was then discarded, while cells were rinsed with PBS before being replenished with fresh media. An MTT assay was employed for the detection of cell viability and proliferation. Briefly, 20 µL of a 0.5% 5 mg/mL MTT reagent was added to each plate, followed by 1–5 h incubation. An ELISA microplate reader was used to assess the O.D. at 570 nm, using an ELISA reader after formazan crystals solubilization with DMSO.

### 3.8. In Silico Studies

#### 3.8.1. Virtual Target Identification

The putative target characterization of Forskolin was achieved via Pharmacophore-based virtual screening using PharmMapper [39]. This platform assigns a score to each molecule in the PDB that best fits a pharmacophore model that has been extracted and stored as a library of ligand datasets in MOL2 format. After that, when a new molecule is submitted, its fit score is calculated for each pharmacophore, and then each fit score for that pharmacophore is compared to the fit score matrix in order to determine where it falls on the scale of all the pharmacophore scores. In comparison to chance pharmacophore matching, the pure fit score that results from this procedure carries considerably more weight and assurance. Before running the virtual screening on the PharmMapper server, the three-dimensional structure of Forskolin was prepared using ChemBio3D, version 12.0 [40]. We chose this software due to its robust molecular modeling capabilities and its acceptance in the scientific community for accurate molecular structure representation. After the initial modeling in ChemBio3D, we recognized the importance of representing the molecule in its most energetically favorable conformation. To this end, we performed a critical step of geometry optimization, which is essential to accurately depict the molecule in its lowest energy state. This step is not just a procedural formality but is crucial for ensuring the reliability of the pharmacophore mapping and subsequent target identification. For the geometry optimization of Forskolin, we utilized the Merck Molecular Force Field 94 (MMFF94) protocol. The MMFF94 protocol is widely acknowledged for its effectiveness in accurately minimizing the energy of a broad range of organic molecules. By employing this protocol, we achieved an optimized structure that closely represents the actual conformation of Forskolin in a biological context. The modeled structure of Forskolin was then submitted to the platform in the MOL2 format using the default settings provided by the PharmMapper server, and then the retrieved results were exported as an Excel sheet, arranging the resulting protein targets according to their fit scores.

#### 3.8.2. Docking Studies

The crystal structures of Cathepsin L (PDB ID: 3BC3; http://www.rcsb.org, accessed on 15 September 2023) were used for the docking study using AutoDock Vina [41]. We utilized a semiflexible docking approach, where the ligand is flexible, while the protein is rigid [42]. The structure of Cathepsin L was fixed to the crystallographic coordinates. The co-crystallized ligand was used to determine the binding site and the docking grid-box in each protein structure, respectively. The co-ordinates of the grid-box were set to be x = 3.73, y = 27.15, and z = 24.1. The ligand–binding site shape matching root means square (RMSD) threshold was set to 2.0 Å. The interaction energies were determined using the Charmm force field (v.1.02) with 10.0 Å as a non-bonded cutoff distance and distance-dependent dielectric. Then, 5.0 Å was set as an energy grid extending from the binding site. Exhaustiveness was set to be 24. Ten poses were generated for each docking experiment. The tested compound, Forskolin, was energy-minimized inside the selected binding pocket. The editing and visualization of the generated binding poses were performed using Pymol 2.5.0. software.

#### 3.8.3. Molecular Dynamics Simulation

NAMD 3.0.0. software was used to perform MD simulation [43,44]. This software applies the Charmm-36 force field. Protein systems were built using the QwikMD toolkit of the VMD software 1.9.3. [44,45], where the protein structures were checked for any missing hydrogens, the protonation states of the amino acid residues were set (pH = 7.4), and the co-crystalized water molecules were removed. Thereafter, the whole structures were embedded in an orthorhombic box of TIP3P water, together with 0.15 M Na^+^ and Cl^−^ ions in 20 Å^3^ solvent buffer. Afterward, the prepared systems were energy-minimized and equilibrated for 5 ns. The whole system was allowed to relax. The production step was set to be either 30 ns for short runs or 200 ns for long ones. The parameters and topologies of the ligands were calculated using the VMD plugin Force Field Toolkit (ffTK). Afterward, the generated parameters and topology files were loaded to VMD to readily read the protein–ligand complexes without errors and conduct the simulation steps.

#### 3.8.4. Binding Free Energy Calculations

Molecular Mechanics Poisson-Boltzmann Surface Area (MM-PBSA) embedded in the MMPBSA.py module of AMBER18 was utilized to calculate the binding free energy of the docked complex [46]. One-hundred frames were processed from the trajectories in total, and the system’s net energy was estimated using the following equation:

ΔG_Binding_ = ΔG_Complex_ − ΔG_Receptor_ − ΔG_Inhibitor_

Each of the aforementioned terms requires the calculation of multiple energy components, including van der Waals energy, electrostatic energy, internal energy from molecular mechanics, and the polar contribution to solvation energy.

## 4. Conclusions

In this study, we embarked on a comprehensive exploration of Forskolin’s antiviral properties, using both in vitro and in silico methodologies. Our investigations revealed that Forskolin exhibits significant antiviral activity against HAV, COX-B4, HSV-1, and HSV-2 with IC_50_ values of 62.9, 73.1, 99.0, and 106.0 μg/mL, respectively, with pharmacophore-based virtual screening identifying Cathepsin L as a primary target. Being a host-dependent molecular target, inhibiting Cathepsin L will provide a broad-spectrum antiviral activity, and hence, this putatively explains its antiviral potential against four different viruses. Molecular docking and dynamic simulations further elucidated the interaction between Forskolin and Cathepsin L, unveiling the role of hydrogen bonds and hydrophobic interactions in stabilizing this binding. These findings not only underscore Forskolin’s potential as a Cathepsin L inhibitor, but also highlight its promising role as an antiviral agent. We acknowledge the pivotal role that the correlation between in silico predictions and experimental results plays in reinforcing the validity of our study. The in silico results laid the foundation for our experimental design, providing a preliminary indication that inhibiting Cathepsin L has the potential to exhibit broad-spectrum antiviral activity. This computational prediction was substantiated by our experimental findings, which demonstrated notable antiviral activity against four different viruses. This congruence not only underscores the robustness of our in silico models but also reinforces our hypothesis about the host-dependent mechanism of Cathepsin L. It is this synergy between the in silico and experimental findings that fortifies our conclusion, positing Cathepsin L inhibition as a potentially effective antiviral strategy. Given these results, Forskolin emerges as a viable candidate for further development in pharmaceutical applications aimed at treating viral infections.

## Figures and Tables

**Table 1:** Antiviral effects (IC_50_) of Forskolin against HSV-1, HSV-2, HAV, and COX-B4. All values are expressed in μg mL^−1^ concentrations.

Compound	IC_50_			
	HSV-1	HSV-2	HAV	COX-B4
Forskolin	99.08	106.01	73.17	62.98

## Data Availability

The data presented in this study are available on request from the corresponding author.

## Supplementary Materials

The following supporting information can be downloaded at: https://www.mdpi.com/article/10.3390/molecules29030704/s1, Figure S1: ^1^H-NMR analysis of C1 (600 MHz, CDCl_3_); Figure S2: ^13^C-NMR analysis of C1 (150 MHz, CDCl_3_); Figure S3: COSY spectrum of C1; Figure S4: NOESY spectrum of C1; Figure S5: HR-ESI-MS spectrum of C1; Figure S6: (a) Dose response analysis representation of the cytotoxic effect of different concentrations of Forskolin for 48 h against Vero cell line growth; (b) Measurement of the efficacy and potency of Forskolin extract with different concentrations as an anti- HSV-1 agent; (c) Measurement of the efficacy and potency of Forskolin extract with different concentrations as an anti- HSV-2 agent; (d) Measurement of the efficacy and potency of Forskolin extract with different concentrations as an anti- CoxB4 agent; (e) Percent of inhibition measurement of HAV exposed to four concentrations of Forskolin extract; Figure S7: The energy-minimized Forskolin structure that was used for the virtual screening and docking experiments as well as the MD simulations; Figure S8: The generated 10 binding poses of Forskolin inside the active site of Cathepsin L (PDB ID 3BC3). The 10 generated poses were almost of the same orientations with slight differences. They occupied the S1 and S1′ subunits of the Cathepsin L’s active site. The RMSD between the best-scoring pose the worst-scoring one was 3.2 Å; Table S1: ^1^H and ^13^C-NMR Spectral Data of Forskolin; Table S2: Cytotoxic activity of Vero cell line exposed to different concentrations of Forskolin for 48 h. The results are presented as percentage (mean ± SD). Cell viability was evaluated by MMT assay; Table S3: Measurement of the efficacy and potency of Forskolin extract with different concentrations as an anti- HSV-1 agent; Table S4: Measurement of the efficacy and potency of Forskolin extract with different concentrations as an anti- HSV-2 agent; Table S5: Measurement of the efficacy and potency of Forskolin extract with different concentrations as an anti- CoxB4 agent; Table S6: Percent of inhibition measurement of HAV exposed to four concentrations of Forskolin extract; Table S7. The PharmMapper results ranked from the highest to the lowest protein hits in terms of Fit-scores; Table S8. The docking scores, binding free energies (Δ*G*_Binding_) in kcal/mol, and the average RMSDs in Å of the generated 10 poses of Forskolin.
