# Supplementary material for: Unveiling the Antiviral Efficacy of Forskolin: A Multifaceted In Vitro and In Silico Approach
Source: Molecules. 2024 Feb 3;29(3):704. doi: 10.3390/molecules29030704 (PMC10856047; doi:10.3390/molecules29030704)
Supplement: Supplementary file 1 [file molecules-29-00704-s001.zip › molecules-2831433-supplementary.pdf]

Supplementary Material

Unveiling the Antiviral Efficacy of Forskolin: A Multifaceted In Vitro and In Silico Approach

Yhiya Amen [1,*], Mohamed A Selim [2], Reda A. Suef [2], Ahmed M. Sayed [3,4], Ahmed Othman [5,*]

[1] Department of Pharmacognosy, Faculty of Pharmacy, Mansoura University, Mansoura 35516, Egypt

[2] Botany and Microbiology Department, Faculty of Science, Al-Azhar University, Cairo 11884, Egypt; mohamedselim@azhar.edu.eg (M.A.S.); redasuef@azhar.edu.eg (R.A.S.)

[3] Department of Pharmacognosy, Collage of Pharmacy, Almaaqal University, Basra 61014, Iraq; ahmed.mohamed.sayed@nub.edu.eg

[4] Department of Pharmacognosy, Faculty of Pharmacy, Nahda University, Beni-Suef 62513, Egypt

[5] Department of Pharmacognosy and Medicinal Plants, Faculty of Pharmacy, Al-Azhar University, Cairo 11884, Egypt

* Correspondence: yhiaamen@mans.edu.eg (Y.A.); ah.othman@azhar.edu.eg (A.O.)

Abstract: *Coleus forskohlii* (Willd.) Briq. is a medicinal herb of the Lamiaceae family. It is native to India and widely present in tropical and sub-tropical regions of Egypt, China, Ethiopia, and Pakistan. The roots of *C. forskohlii* are edible, rich with pharmaceutically bioactive compounds, and traditionally reported to treat a variety of diseases, including inflammation, respiratory disorders, obesity, and viral ailments. Notably, the emergence of viral diseases is expected to quickly spread; consequently, these data impose a need for various approaches to develop broad active therapeutics for utilization in the management of future viral infection outbreaks. In this study, the naturally occurring labdane diterpenoid derivative, forskolin, was obtained from *Coleus forskohlii*. Additionally, we evaluated the antiviral potential of forskolin towards three viruses, namely herpes simplex viruses 1 and 2 (HSV-1 and HSV-2), hepatitis A virus (HAV), and coxsackievirus B4 (COX-B4). We observed that forskolin displayed antiviral activity against HAV, COX-B4, HSV-1, HSV-2 with IC_{50} values of 62.9, 73.1, 99.0, and 106.0 μg/mL, respectively. Furthermore, we explored the forskolin's potential antiviral target using PharmMapper, a pharmacophore-based virtual screening platform. Forskolin's modeled structure was analyzed to identify potential protein targets linked to its antiviral activity, with results ranked based on Fit scores. Cathepsin L (PDB ID: 3BC3) emerged as a top-scoring hit, prompting further exploration through molecular docking and MD simulations. Our analysis revealed that forskolin's binding mode within cathepsin L's active site, characterized by stable hydrogen bonding and hydrophobic interactions, mirrors that of a co-crystallized inhibitor. These findings, supported by consistent RMSD profiles and similar binding free energies, suggest forskolin's potential in inhibiting cathepsin L, highlighting its promise as an antiviral agent.

Keywords: *Coleus forskohlii*; Forskolin; HAV; COX-B4; HSV-1; HSV-2; Virtual screening, MD simulation, Cathepsin L.

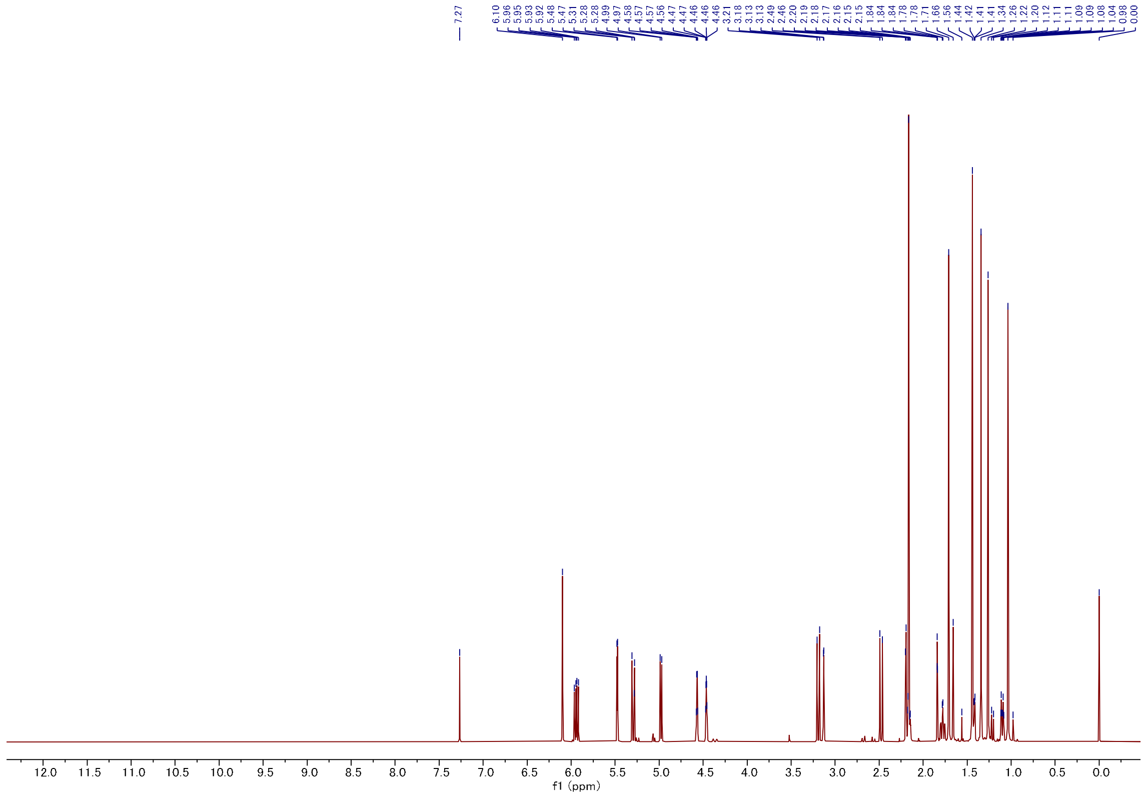

Figure S1: ^{1}H-NMR analysis of C1 (600 MHz, $CDCl_3$)

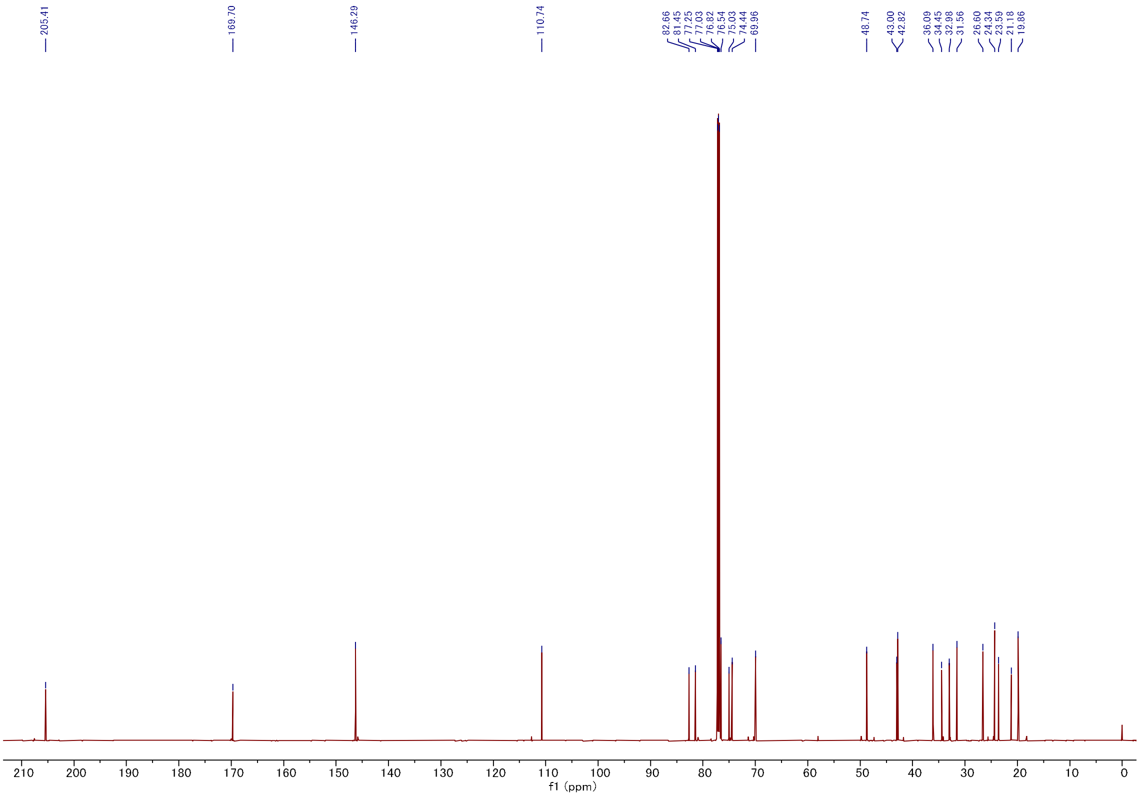

Figure S2: ^{13}C-NMR analysis of C1 (150 MHz, $CDCl_3$)

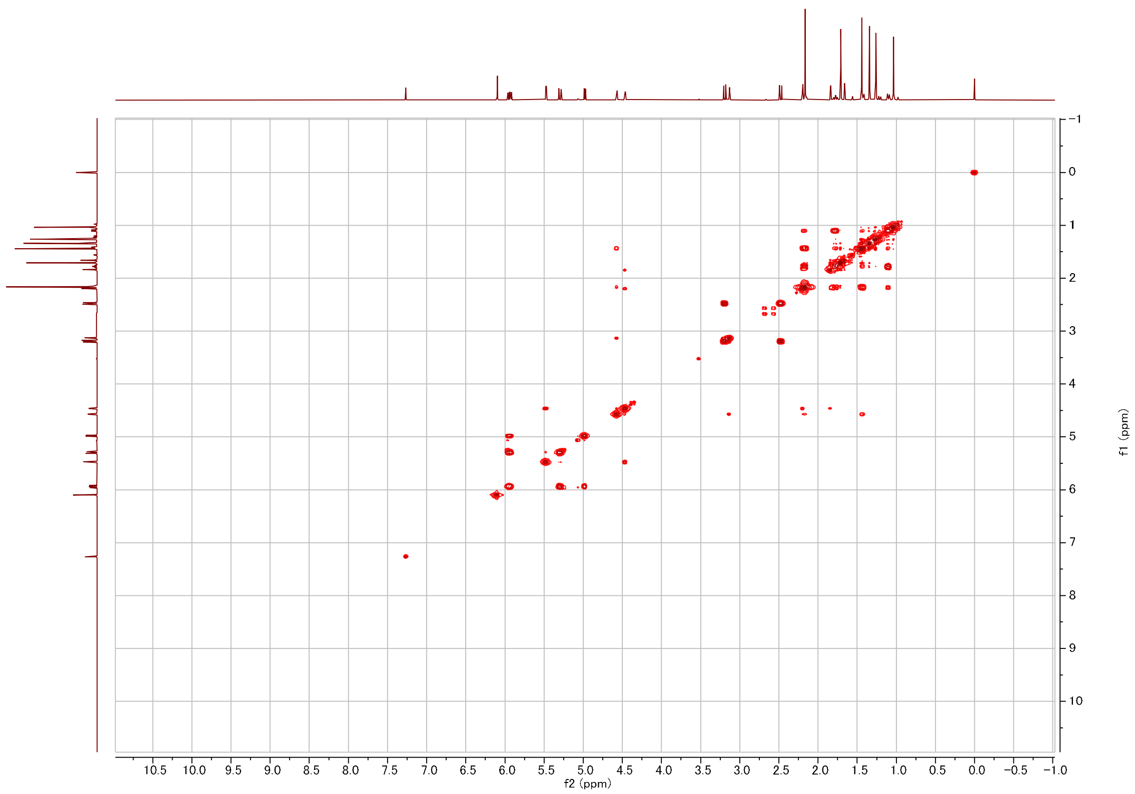

Figure S3: COSY spectrum of C1

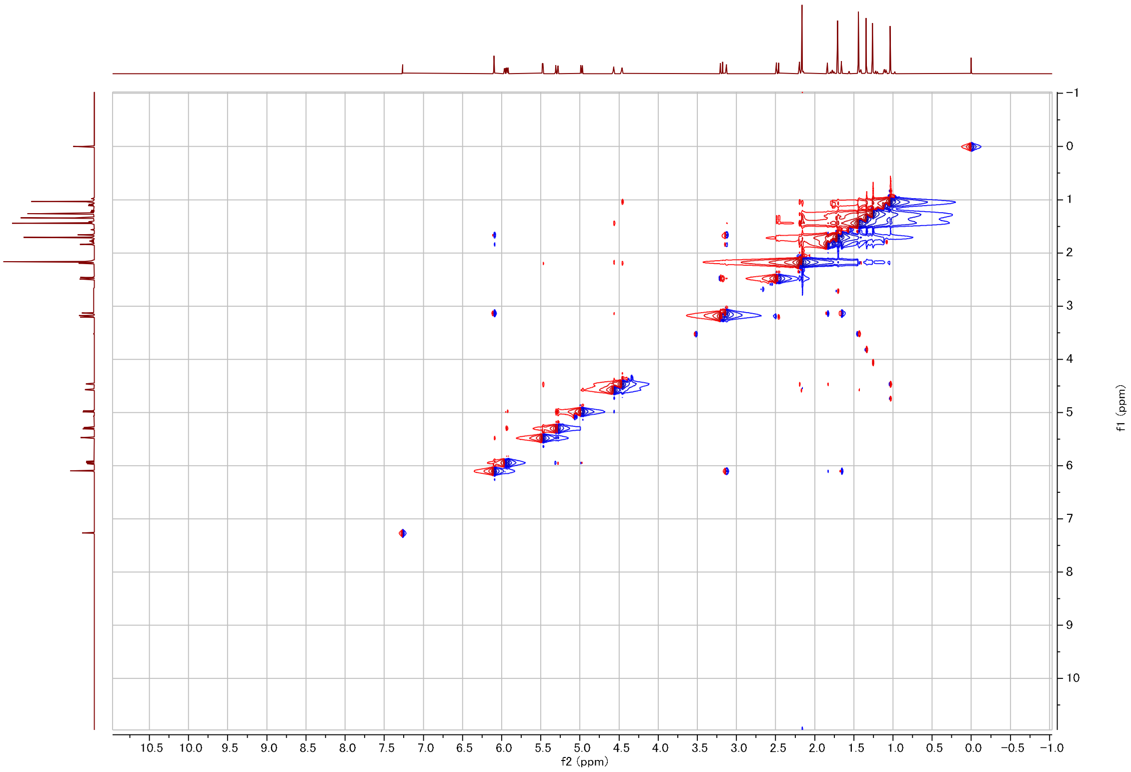

Figure S4: NOESY spectrum of C1

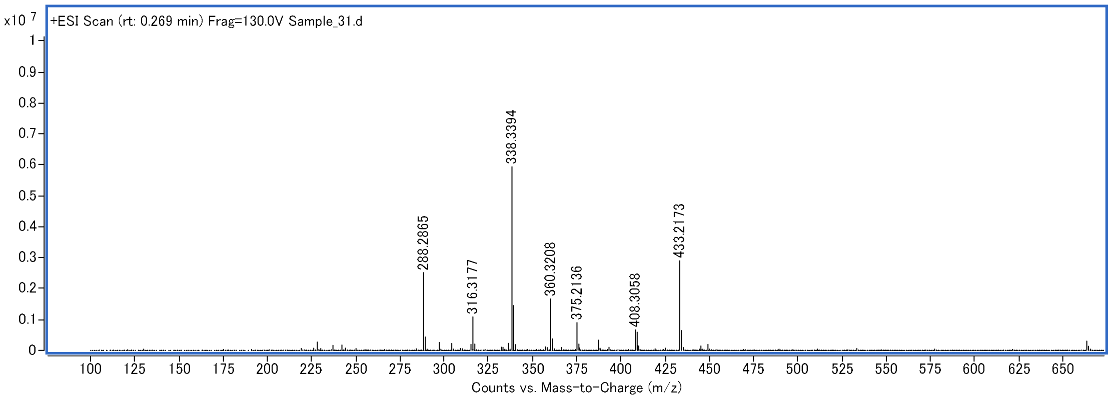

Figure S5: HR-ESI-MS spectrum of C1

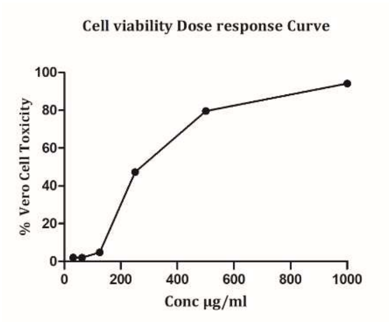

(**a**)

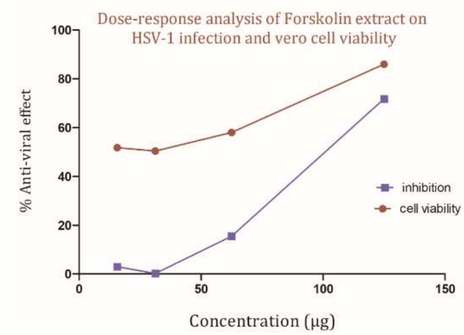

(**b**)

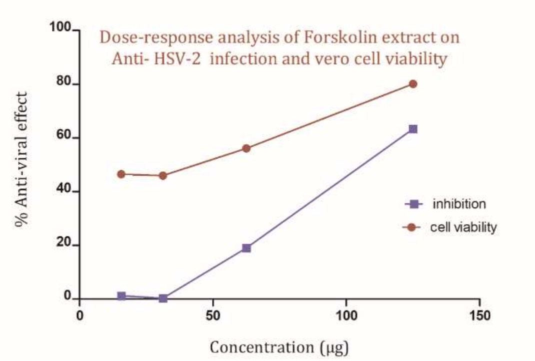

(c)

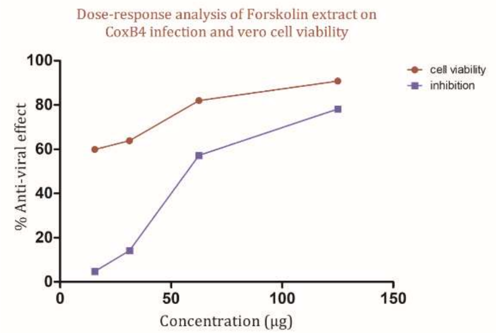

(d)

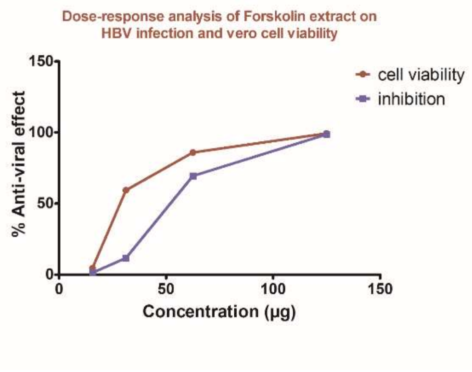

(e)

Figure S6: (a) Dose response analysis representation of the cytotoxic effect of different concentrations of Forskolin for 48 hour against Vero cell line growth; (b) Measurement of the efficacy and potency of forskolin extract with different concentrations as an anti- HSV-1 agent; (c) Measurement of the efficacy and potency of forskolin extract with different concentrations as an anti- HSV-2 agent; (d) Measurement of the efficacy and potency of forskolin extract with different concentrations as an anti- CoxB4 agent; (e) Percent of inhibition measurement of HAV exposed to four concentrations of forskolin extract.

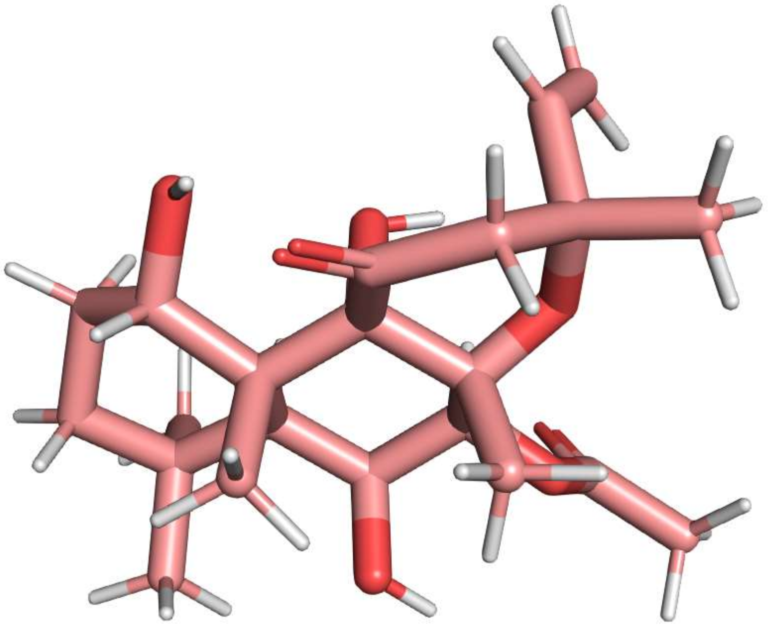

Figure S7. The energy-minimized forskolin structure that was used for the virtual screening and docking experiments as well as the MD simulations.

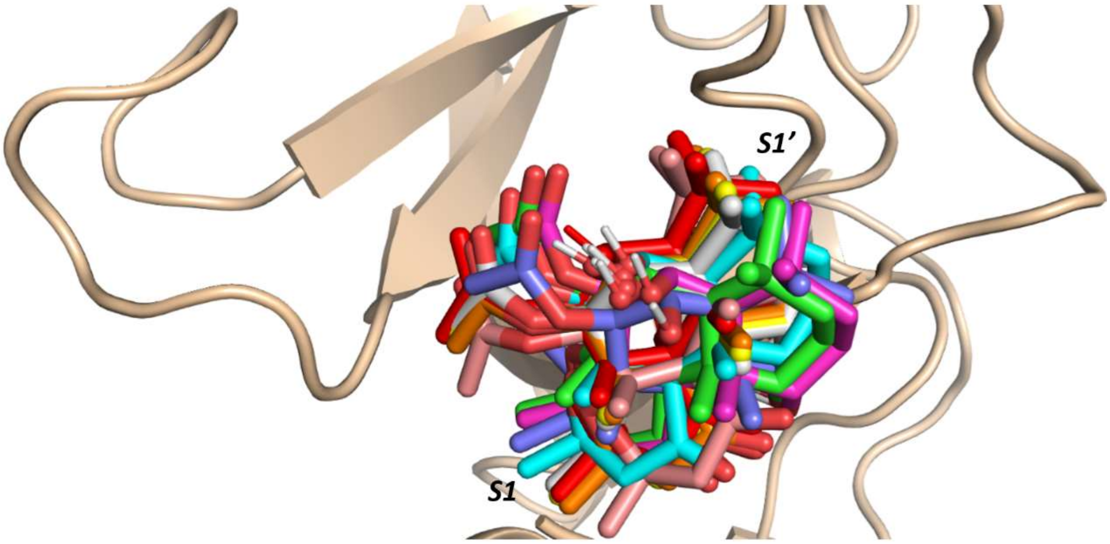

Figure S8. The generated 10 binding poses of forskolin inside the active site of Cathepsin L (PDB ID 3BC3). The 10 generated poses were almost of the same orientations with slight differences. They occupied the S1 and S1′ subunits of the Cathepsin L′s active site. The RMSD between the best-scoring pose the worst-scoring one was 3.2 Å.

Table S1. ^{1}H and ^{13}C-NMR Spectral Data of Forskolin.

#	**^{1}H** (multiplicity, *J* in Hz)	**^{13}C**
1		74.4
2		26.6
3		36.1
4		34.5
5		42.8
6	4.5 (1H, brs)	70.0
7	5.28 (1H, d, *J* = 16.3)	76.5
8		81.5
9		82.7
10		43.0
11		205.4
12		48.7
13		75.0
14	5.87 (1H, dd, *J* = 10.6, 17.2 Hz)	146.3
15	4.91 (1H, d, *J* = 10.9)	110.7
	5.40 (1H, d, *J* = 17.2)	
16	1.34	31.6
17	1.71	23.6
18	1.04	33.0
19	1.26	24.3
20	1.44	19.9
21		169.7
22	2.16	21.2

Table S2: Cytotoxic activity of Vero cell line exposed to different concentrations of Forskolin for 48 h. The results are presented as percentage (mean ± SD). Cell viability was evaluated by MMT assay.

Plant extract		Cell proliferation (Cytotoxicity % VS Control)						
		Vero Cell Line						
**Conc μg/ml**	**Denotation**	**Control**	**1000**	**500**	**250**	**125**	**62.5**	**31.25**
**Forskolin**	**Mean O.D**	0.7	0.04	0.14	0.37	0.66	0.68	0.68
	**± SD**	0	0	0.01	0.01	0.01	0	0.01
	**% Viability**	100	5.8	20.38	52.71	95.25	98.03	97.99
	**% Toxicity**	**0**	94.2	79.62	47.29	4.75	1.97	2.01
	**CC_{50} ±SD**	**322.1 ± 9.58**						
	**MNTC μg/ml**	**125**						

Table S3: Measurement of the efficacy and potency of forskolin extract with different concentrations as an anti-**HSV-1** agent.

Plant extract		Cell	virus	Anti- HSV-1 Effect of Forskolin			
**Conc µg/ml**	**Denotation**	**Vero**	**HSV-1**	**125**	**62.5**	**31.25**	**15.62**
Forskolin	**Mean O.D**	0.71	0.36	0.61	0.41	0.36	0.37
	**± SD**	0	0	0.01	0.01	0	0
	**% Viability**	100	50.28	85.98	57.98	50.38	51.75
	**% Toxicity**	-	49.72	14.02	42.02	49.62	48.25
	**% HSV-1 Activity**	-	100	28.21	84.52	99.81	97.06
	**% Anti-viral effect**	-	0	71.79	15.48	0.19	2.94
	**IC_{50} ±SD**			**99.083**			

Table S4: Measurement of the efficacy and potency of forskolin extract with different concentrations as an anti-**HSV-2** agent.

Plant extract		Cell	virus	Anti- HSV-2 Effect of Forskolin			
**Conc µg/ml**	**Denotation**	**Vero**	**HSV-2**	**125**	**62.5**	**31.25**	**15.62**
Forskolin	**Mean O.D**	0.71	0.36	0.57	0.4	0.32	0.33
	**± SD**	0	0	0	0	0	0
	**% Viability**	100	50.28	80.12	56.09	45.94	46.46
	**% Toxicity**	-	49.72	19.88	43.91	54.06	53.54
	**% HSV-1 Activity**	-	100	36.67	81.01	99.74	98.78
	**% Anti-viral effect**	-	0	63.33	18.99	0.26	1.22
	**IC_{50} ±SD**			**106.01**			

Table S5: Measurement of the efficacy and potency of forskolin extract with different concentrations as an anti-**CoxB4** agent.

Plant extract		Cell	virus	Anti- CoxB4 Effect of Forskolin			
**Conc µg/ml**	**Denotation**	**Vero**	**CoxB4**	**125**	**62.5**	**31.25**	**15.62**
	**Mean O.D**	0.71	0.41	0.64	0.58	0.45	0.42
	**± SD**	0	0.01	0.01	0.01	0.01	0
**Forskolin**	**% Viability**	100	57.88	90.79	81.96	63.83	59.87
	**% Toxicity**	-	42.12	9.21	18.04	36.17	40.13
	**% HSV-1 Activity**		100	21.86	42.83	85.87	95.29
	**% Anti-viral effect**		**0**	78.14	57.17	14.13	4.71
	**IC_{50} ±SD**			**73.17**			

Table S6: Percent of inhibition measurement of HAV exposed to four concentrations of forskolin extract.

Plant extract		Cell	virus	Anti- HAV Effect of Forskolin			
**Conc μg/ml**	**Denotation**	**Vero**	**HAV**	**125**	**62.5**	**31.25**	**15.62**
**Forskolin**	**Mean O.D**	0.71	0.38	0.70	0.61	0.42	0.39
	**± SD**	0	0.01	0.01	0.01	0.01	0.00
	**% Viability**	100	53.9	99.34	85.93	59.35	4.58
	**% Toxicity**	0	46.03	0.66	14.07	40.65	5.42
	**% HSV-1 Activity**	-	100	1.44	30.56	88.31	98.67
	**% Anti-viral effect**	-	0	98.66	69.44	11.69	1.33
	**IC_{50} ±SD**			**62.986**			

Table S7. The PharmMapper results ranked from the highest to the lowest protein hits in terms of Fit-scores.

Rank	PDB ID	Fit-Score	Target Name
1	2ct7	19.46	RING finger protein 31
2	1evy	17.86	Glycerol-3-phosphate dehydrogenase [NAD+], glycosomal
3	3bc3	16.81	S subsites of cathepsin L
4	1roz	14.59	Deoxyhypusine synthase
5	1f9u	14.39	Kinesin-like protein KAR3
6	1n8w	14.19	Malate synthase G
7	1n8z	13.79	Receptor tyrosine-protein kinase erbB-2
8	2ify	12.58	2,3-bisphosphoglycerate-independent phosphoglycerate mutase
9	1uyr	12.2	Acetyl-CoA carboxylase
10	2gqf	11.85	Uncharacterized protein HI0933
11	2yxr	11.77	Preprotein translocase subunit secY
12	2c36	11.76	Glycoprotein D
13	2e8x	11.69	Geranylgeranyl pyrophosphate synthetase
14	2i15	11.47	Uncharacterized protein MG296 homolog
15	1g7s	10.71	Probable translation initiation factor IF-2
16	1w1g	10.7	3-phosphoinositide-dependent protein kinase 1
17	3g7g	10.7	UPF0311 protein CA_C3321
18	1sh4	10.44	Cytochrome b5
19	2hz7	10.38	Glutaminyl-tRNA synthetase
20	1wvc	10.36	Glucose-1-phosphate cytidylyltransferase
21	1qrj	10.23	Gag-Pro-Pol polyprotein
22	3dwl	10.23	Actin-related protein 3
23	1nda	10.15	Trypanothione reductase

24	1w5a	9.926	Cell division protein ftsZ homolog 1
25	3ffv	9.857	Protein syd
26	1lj0	9.748	Cytochrome b5 type B
27	2nr0	9.445	tRNA pseudouridine synthase A
28	1ujv	9.415	Membrane-associated guanylate kinase, WW and PDZ domain-containing protein 2
29	2ix4	9.292	3-oxoacyl-[acyl-carrier-protein] synthase, mitochondrial
30	3fqd	9.037	5-3 exoribonuclease 2
31	1fi6	8.889	RalBP1-associated Eps domain-containing protein 1
32	1sfk	8.882	Genome polyprotein
33	1ddm	8.824	Protein numb
34	1iq3	8.736	RalBP1-associated Eps domain-containing protein 2
35	2j96	8.712	Phycoerythrocyanin alpha chain
36	1oxy	8.694	Hemocyanin II
37	3esw	8.495	Peptide-N(4)-(N-acetyl-beta-glucosaminyl)asparagine amidase
38	1tg6	8.493	Putative ATP-dependent Clp protease proteolytic subunit, mitochondrial
39	2e3x	8.4	Coagulation factor X-activating enzyme heavy chain
40	1x65	8.276	Cold shock domain-containing protein E1
41	1jv1	8.223	UDP-N-acetylhexosamine pyrophosphorylase
42	3b6x	8.212	General odorant-binding protein lush
43	2uvn	8.211	Putative cytochrome P450 130
44	1k44	8.2	Nucleoside diphosphate kinase
45	3gh8	8.177	Iodotyrosine dehalogenase 1
46	1l6s	8.148	Delta-aminolevulinic acid dehydratase
47	1ya0	8.094	Protein SMG7
48	1rso	8.091	Disks large homolog 1

49	3d3l	8.091	Arachidonate 12-lipoxygenase, 12S-type
50	1zte	8.07	Superoxide dismutase [Mn], mitochondrial
51	1xjv	8.054	Protection of telomeres protein 1
52	2ibm	8.041	Protein translocase subunit secA
53	1r8u	8.041	Cbp/p300-interacting transactivator 2
54	1zag	7.992	Zinc-alpha-2-glycoprotein
55	1in7	7.976	Holliday junction ATP-dependent DNA helicase ruvB
56	1nsh	7.957	Protein S100-A11
57	1hm6	7.956	Annexin A1
58	1ako	7.956	Exodeoxyribonuclease III
59	1p7o	7.933	Phospholipase A2, acidic 2
60	1cok	7.927	Tumor protein p73
61	2dld	7.885	D-lactate dehydrogenase
62	2ior	7.866	Chaperone protein htpG
63	1x1f	7.865	Signal-transducing adaptor protein 1
64	2p54	7.86	Peroxisome proliferator-activated receptor alpha
65	1gm6	7.853	Salivary lipocalin
66	2r0n	7.846	Glutaryl-CoA dehydrogenase, mitochondrial
67	1tnq	7.832	Troponin C, skeletal muscle
68	1snl	7.817	Nucleobindin-1
69	2csw	7.813	E3 ubiquitin-protein ligase RNF8
70	1na6	7.769	Type-2 restriction enzyme EcoRII
71	3ibv	7.76	Exportin-T
72	2nq5	7.758	5-methyltetrahydropteroyltriglutamate--homocysteine methyltransferase
73	2yww	7.758	Aspartate carbamoyltransferase regulatory chain
74	1yc9	7.753	Multidrug resistance protein, putative
75	1wde	7.734	Probable diphthine synthase

76	2h63	7.71	Biliverdin reductase A
77	3bch	7.706	40S ribosomal protein SA
78	2b61	7.704	Homoserine O-acetyltransferase
79	1win	7.682	Flotillin-2
80	2jpe	7.68	Nuclear inhibitor of protein phosphatase 1
81	1b7a	7.678	Phosphatidylethanolamine-binding protein 1
82	1ay5	7.672	Aromatic-amino-acid aminotransferase
83	2qby	7.65	Cell division control protein 6 homolog 1
84	3eap	7.638	Rho GTPase-activating protein 11A
85	1civ	7.625	Malate dehydrogenase [NADP], chloroplast
86	1vrn	7.624	Photosynthetic reaction center cytochrome c subunit
87	1b8h	7.613	DNA polymerase processivity component
88	3df0	7.606	Calpain-2 catalytic subunit
89	1xvv	7.58	Crotonobetainyl-CoA:carnitine CoA-transferase
90	1vl7	7.546	Alr5027 protein
91	2gfp	7.539	Multidrug resistance protein D
92	1ggt	7.53	Coagulation factor XIII A chain
93	1m0t	7.514	Glutathione synthetase
94	1s5a	7.51	Hypothetical protein yesE
95	1a79	7.507	tRNA-splicing endonuclease
96	1dc1	7.448	Type-2 restriction enzyme BsoBI
97	2gp6	7.444	3-oxoacyl-[acyl-carrier-protein] synthase 2
98	1a0h	7.433	Prothrombin
99	1zun	7.43	Sulfate adenylyltransferase subunit 2
100	1uv6	7.422	Acetylcholine-binding protein
101	1ykd	7.416	Adenylate cyclase
102	1gzq	7.406	T-cell surface glycoprotein CD1b
103	1b0a	7.405	Bifunctional protein folD

104	1fiz	7.404	Acrosin
105	1ahj	7.394	Nitrile hydratase subunit alpha
106	2vw5	7.374	ATP-dependent molecular chaperone HSP82
107	2cpt	7.37	Vacuolar protein sorting-associated protein 4B
108	2hcb	7.368	Chromosomal replication initiator protein dnaA
109	2qtv	7.351	Protein transport protein SEC23
110	1p32	7.348	Complement component 1 Q subcomponent-binding protein, mitochondrial
111	2hvb	7.347	Superoxide reductase
112	3eay	7.345	Sentrin-specific protease 7
113	1r2j	7.343	FkbI
114	2qxl	7.34	Heat shock protein homolog SSE1
115	2bjh	7.335	Feruloyl esterase A
116	2czy	7.332	Paired amphipathic helix protein Sin3b
117	1vap	7.327	Phospholipase A2
118	2gsq	7.324	Glutathione S-transferase
119	1ow1	7.322	Msx2-interacting protein
120	1lrw	7.32	Methanol dehydrogenase subunit 1
121	3epm	7.319	Thiamine biosynthesis protein thiC
122	1asq	7.316	L-ascorbate oxidase
123	1rp1	7.316	Pancreatic lipase-related protein 1
124	1ix1	7.31	Peptide deformylase
125	1aj8	7.295	Citrate synthase
126	2j47	7.292	O-GlcNAcase BT_4395
127	1wxr	7.286	Hemoglobin-binding protease hbp
128	2.00E+55	7.277	Uracil phosphoribosyltransferase
129	1lnl	7.272	Hemocyanin type 2 unit e
130	1q1h	7.272	Transcription factor E

131	2k8y	7.27	Uncharacterized protein MJ0187
132	1wh0	7.267	Ubiquitin carboxyl-terminal hydrolase 19
133	1wru	7.266	Baseplate protein
134	1k1d	7.264	D-hydantoinase
135	1vcn	7.261	CTP synthase
136	1spu	7.261	Primary amine oxidase
137	3dra	7.248	Protein farnesyltransferase/geranylgeranyltransferase type-1 subunit alpha
138	2a98	7.246	Inositol-trisphosphate 3-kinase C
139	1z45	7.241	GAL10 bifunctional protein [Includes: UDP-glucose 4-epimerase
140	1t90	7.24	Methylmalonate semialdehyde dehydrogenase [acylating]
141	1z9f	7.238	Single-stranded DNA-binding protein
142	2ikq	7.234	Ubiquitin-associated and SH3 domain-containing protein B
143	1qe0	7.233	Histidyl-tRNA synthetase
144	1d5y	7.233	Right origin-binding protein
145	1sq1	7.233	Chorismate synthase
146	2qv6	7.231	GTP cyclohydrolase III
147	1uh6	7.227	Ubiquitin-like protein 5
148	1k9k	7.227	Protein S100-A6
149	1m1j	7.224	Fibrinogen alpha chain
150	1j3x	7.224	High mobility group protein B2
151	1qwo	7.222	3-phytase A
152	1xge	7.218	Dihydroorotase
153	1i11	7.218	Transcription factor SOX-5
154	1fy7	7.214	Histone acetyltransferase ESA1
155	1vhd	7.212	Alcohol dehydrogenase, iron-containing

156	2ee4	7.203	Rho GTPase-activating protein 5
157	1hty	7.203	Alpha-mannosidase 2
158	2f66	7.203	Suppressor protein STP22 of temperature-sensitive alpha-factor receptor and arginine permease
159	2a9e	7.202	Catalase
160	2daf	7.183	IQ and ubiquitin-like domain-containing protein
161	1j7x	7.182	Retinol-binding protein 3
162	1esj	7.179	Hydroxyethylthiazole kinase

Table S8. The docking scores, binding free energies ($\Delta G_{Binding}$) in kcal/mol, and the average RMSDs in Å of the generated 10 poses of forskolin.

Pose number	Docking score	$\Delta G_{Binding}$*	average RMSD#
1	-10.37	-8.33	1.87
2	-9.78	-8.16	1.97
3	-9.63	-7.68	2.47
4	-9.61	-7.37	2.59
5	-9.48	-7.44	2.69
6	-9.32	-7.28	2.63
7	-9.28	-7.21	2.91
8	-9.21	-7.17	2.46
9	-9.18	-7.15	2.28
10	-9.11	-7.18	2.23

* and # the calculated binding free energies and RMSD were based on 30 ns-long MD simulations
